# 1-[3,5-Bis(4-fluoro­phen­yl)-4,5-dihydro-1*H*-pyrazol-1-yl]ethanone

**DOI:** 10.1107/S1600536810004435

**Published:** 2010-02-10

**Authors:** Hoong-Kun Fun, Madhukar Hemamalini, S. Samshuddin, B. Narayana, H. S. Yathirajan

**Affiliations:** a X-ray Crystallography Unit, School of Physics, Universiti Sains Malaysia, 11800 USM, Penang, Malaysia; b Department of Studies in Chemistry, Mangalore University, Mangalagangotri 574 199, India; c Department of Studies in Chemistry, University of Mysore, Mysore 570 006, India

## Abstract

In the asymmetric unit of the title compound, C_17_H_14_F_2_N_2_O, there are three independent mol­ecules (*A*, *B* and *C*) which differ slightly in the relative orientations of the two fluoro­phenyl rings. In mol­ecules *A* and *C* one of the fluoro­phenyl rings is disordered over two positions, with occupancy ratios of 0.72 (2):0.28 (2) for mol­ecule *A* and 0.67 (2):0.33 (2) for mol­ecule *C*. The dihedral angle between the two fluoro­phenyl rings in the independent mol­ecules lie in the range 70.3 (3)–84.0 (3)°. In the crystal structure, the mol­ecules are linked *via* inter­molecular C—H⋯O and C—H⋯F hydrogen bonds and π⋯π stacking inter­actions [centroid–centroid distance = 3.7508 (13) Å], forming a three-dimensional network.

## Related literature

For the biological activity of pyrazoline derivatives, see: Amir *et al.* (2008 ▶); Garge *et al.* (1971 ▶); Hes *et al.* (1978 ▶); Manna *et al.* (2005 ▶); Regaila *et al.* (1979 ▶). For the role of pyrazolines in organic synthesis, see: Bhaskarreddy *et al.* (1997 ▶); Klimova *et al.* (1999 ▶). For related structures, see: Anuradha *et al.* (2008 ▶); Jian *et al.* (2006 ▶); Jian & Wang (2006 ▶); Lu *et al.* (2008 ▶); Wang *et al.* (2005 ▶). For the stability of the temperature controller used in the data collection, see: Cosier & Glazer (1986 ▶).
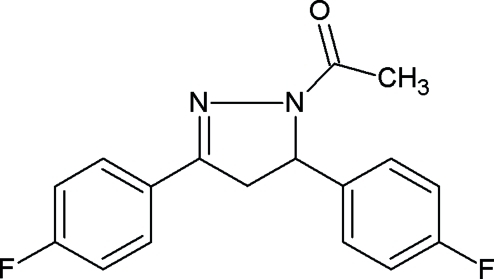


## Experimental

### 

#### Crystal data

C_17_H_14_F_2_N_2_O
*M* *_r_* = 300.30
Triclinic, 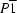

*a* = 7.1447 (1) Å
*b* = 17.2332 (3) Å
*c* = 18.4871 (4) Å
α = 102.880 (1)°
β = 97.941 (1)°
γ = 96.373 (1)°
*V* = 2173.86 (7) Å^3^
*Z* = 6
Mo *K*α radiation
μ = 0.11 mm^−1^
*T* = 100 K
0.40 × 0.27 × 0.21 mm

#### Data collection

Bruker SMART APEXII CCD area-detector diffractometer
Absorption correction: multi-scan (*SADABS*; Bruker, 2009 ▶) *T*_min_ = 0.960, *T*_max_ = 0.978
40717 measured reflections
12544 independent reflections
6078 reflections with *I* > 2σ(*I*)
*R*_int_ = 0.039

#### Refinement

*R*[*F* ^2^ > 2σ(*F* ^2^)] = 0.078
*wR*(*F* ^2^) = 0.242
*S* = 1.03
12544 reflections
726 parameters
510 restraints
H-atom parameters constrained
Δρ_max_ = 0.32 e Å^−3^
Δρ_min_ = −0.22 e Å^−3^

### 

Data collection: *APEX2* (Bruker, 2009 ▶); cell refinement: *SAINT* (Bruker, 2009 ▶); data reduction: *SAINT*; program(s) used to solve structure: *SHELXTL* (Sheldrick, 2008 ▶); program(s) used to refine structure: *SHELXTL*; molecular graphics: *SHELXTL*; software used to prepare material for publication: *SHELXTL* and *PLATON* (Spek, 2009 ▶).

## Supplementary Material

Crystal structure: contains datablocks global, I. DOI: 10.1107/S1600536810004435/ci5026sup1.cif

Structure factors: contains datablocks I. DOI: 10.1107/S1600536810004435/ci5026Isup2.hkl

Additional supplementary materials:  crystallographic information; 3D view; checkCIF report

## Figures and Tables

**Table 1:** Hydrogen-bond geometry (Å, °)

*D*—H⋯*A*	*D*—H	H⋯*A*	*D*⋯*A*	*D*—H⋯*A*
C12*A*—H12*A*⋯F2*A*^i^	0.93	2.53	3.294 (3)	139
C12*B*—H12*B*⋯F2*C*^ii^	0.93	2.53	3.309 (3)	141
C12*C*—H12*C*⋯F2*B*^iii^	0.93	2.53	3.320 (3)	143
C17*A*—H17*A*⋯F1*C*^iv^	0.96	2.53	3.276 (8)	134
C17*B*—H17*D*⋯F1*A*^v^	0.96	2.47	3.309 (4)	146
C17*C*—H17*G*⋯F1*B*^i^	0.96	2.55	3.311 (2)	137
C15*A*—H15*A*⋯O1*C*	0.93	2.32	3.225 (3)	165
C15*B*—H15*B*⋯O1*A*^vi^	0.93	2.36	3.262 (3)	162
C15*C*—H15*C*⋯O1*B*^vii^	0.93	2.42	3.281 (3)	153

Symmetry codes: (i) 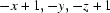; (ii) 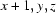; (iii) 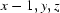; (iv) 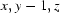; (v) 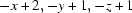; (vi) 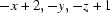; (vii) 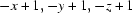.

**Fig. 1.**
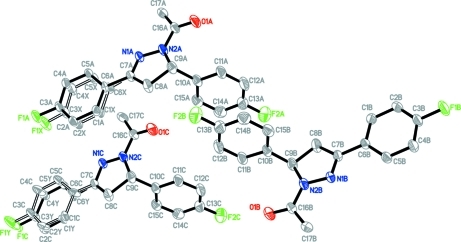
The three independent molecules of the title compound, showing 30% probability displacement ellipsoids and the atom-numbering scheme. Open bonds indicate minor disorder components. H atoms have been omitted for clarity.

**Fig. 2.**
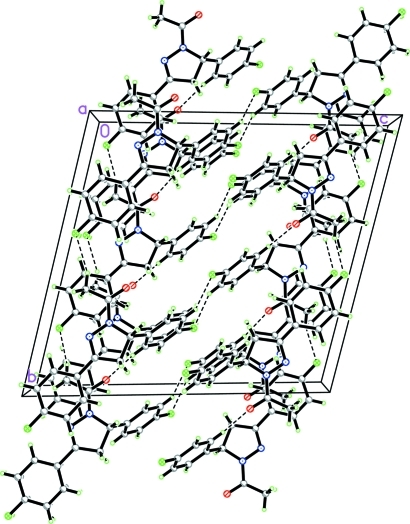
The crystal packing of the title compound, showing a hydrogen-bonded (dashed lines) network.

## supplementary crystallographic information

### Comment

Considerable attention has been focused on substituted pyrazoline compounds, due to their biological activity. In particular, they are used as antitumor, antibacterial, antifungal, antiviral, antiparasitic, anti-tubercular and insecticidal agents (Hes *et al.*, 1978; Manna *et al.*, 2005; Amir *et al.*, 2008). Some of these compounds have also exhibit anti-inflammatory, anti-diabetic, anaesthetic and analgesic properties (Garge *et al.*, 1971; Regaila *et al.*, 1979). Among the pyrazoline derivatives, 1-acetyl-pyrazolines have been identified as one of the most promising scaffold. In the field of medicinal chemistry, 1-acetyl-pyrazoline derivatives have been found to display anticancer and anti-inflammatory activities. In addition, pyrazolines have played a crucial part in the development of the theory of heterocyclic chemistry and are also used extensively in organic synthesis (Klimova *et al.*, 1999; Bhaskarreddy *et al.*, 1997).

The crystal structures of 1-[5-(4-hydroxy-3-methoxyphenyl)-3-methyl-4,5-dihydro-1H-pyrazol-1-yl]ethanone (Wang *et al.*, 2005), 1-[4,5-dihydro-5-phenyl-3-(p-tolyl) pyrazol-1-yl]ethanone (Jian *et al.*, 2006), 1-[3-(4-methoxyphenyl)- 5-phenyl-4,5-dihydro-1H-pyrazol-1-yl]ethanone (Jian & Wang, 2006), 1-[3-(2,4-dichloro-5-fluorophenyl)-5-(3-methyl-2-thienyl)-4,5-dihydro-1H- pyrazol-1-yl]ethanone (Anuradha *et al.*, 2008) and 1-[3-(2-naphthyl)-5-(3,4,5-trimethoxyphenyl)-4,5-dihydro- 1H-pyrazol-1-yl]ethanone (Lu *et al.*, 2008) have been reported. Pyrazolines can be conveniently synthesized by the treatment of α,β-unsaturated carbonyl compounds with hydrazine reagents in basic and acidic media. In this method, hydrazones are formed as intermediates, which can be subsequently cyclized to 2-pyrazolines in the presence of acetic acid. In view of the biological importance of pyrazolines, we report here the crystal structure of a new pyrazoline derivative.

The asymmetric unit of the title compound consists of three crystallographically independent molecules, A, B and C (Fig. 1). In each independent molecule, the pyrazole ring adopts a flattened envelope conformation; the flap atoms C9A, C9B and C9C are displaced by 0.078 (3) and 0.099 (3) and 0.205 (3) Å from the planes of the other four atoms. The dihedral angle between the two fluorophenyl rings are: 70.3 (3) and 72.7 (9)° in the major and minor components of molecule A, 74.73 (10)° in molecule B and 84.0 (3) and 76.1 (9)° in the major and minor components of molecule C.

In the crystal structure (Fig. 2), the molecules are linked through intermolecular C—H···O and C—H···F hydrogen bonds (see Table 1) to form three-dimensional network. The crystal structure is further stabilized by weak π···π stacking interactions between C10A–C15A and C10B–C15B phenyl rings at (x, y, z), with a ring centroid-to-centroid distance of 3.7509 (13)Å.

### Experimental

A mixture of (2E)-1,3-bis(4-fluorophenyl)prop-2-en-1-one (2.44 g, 0.01 mol) and hydrazine hydrate (0.5ml, 0.01 mol) in ethanol (25 ml) in presence of glacial acetic acid (2 ml) was refluxed for 5 h. The reaction mixture was cooled and poured into 50 ml ice-cold water. The precipitate was collected by filtration and purified by recrystallization from ethanol. Single crystals were grown by slow evaporation of a toluene solution (yield: 84%, m.p. 387 K). Analytical data: Found (Calculated): C 67.86 (67.99), H 4.62 (4.70), N 9.29 (9.33)%.

### Refinement

One of the 4-fluorophenyl rings (F1/C1-C6) in molecules A and C are each disordered over two positions with occupancies of 0.72 (2) and 0.28 (2) for molecule A, and 0.67 (2) and 0.33 (2) for molecule C. In all disorder components atoms closer than 1.7 Å were restrained to have the same U^ij^ components and all bonds were subjected to a rigid bond restraint. The 1,2- and 1,3-distances in the major and minor disorder components were restrained to be the same. H atoms were positioned geometrically [C–H = 0.93–0.98 Å] and were refined using a riding model, with *U*_iso_(H) = 1.2 or 1.5 *U*_eq_(C). A rotating group model was used for the methyl groups.

### Crystal data

**Table:**

C_17_H_14_F_2_N_2_O	*Z* = 6
*M**_r_* = 300.30	*F*(000) = 936
Triclinic, *P*1	*D*_x_ = 1.376 Mg m^−^^3^
Hall symbol: -P 1	Mo *K*α radiation, λ = 0.71073 Å
*a* = 7.1447 (1) Å	Cell parameters from 8334 reflections
*b* = 17.2332 (3) Å	θ = 2.9–30.0°
*c* = 18.4871 (4) Å	µ = 0.11 mm^−^^1^
α = 102.880 (1)°	*T* = 100 K
β = 97.941 (1)°	Block, colourless
γ = 96.373 (1)°	0.40 × 0.27 × 0.21 mm
*V* = 2173.86 (7) Å^3^	

### Data collection

**Table:**

Bruker SMART APEXII CCD area-detector diffractometer	12544 independent reflections
Radiation source: fine-focus sealed tube	6078 reflections with *I* > 2σ(*I*)
graphite	*R*_int_ = 0.039
φ and ω scans	θ_max_ = 30.0°, θ_min_ = 1.2°
Absorption correction: multi-scan (*SADABS*; Bruker, 2009)	*h* = −10→10
*T*_min_ = 0.960, *T*_max_ = 0.978	*k* = −22→24
40717 measured reflections	*l* = −20→25

### Refinement

**Table:**

Refinement on *F*^2^	Primary atom site location: structure-invariant direct methods
Least-squares matrix: full	Secondary atom site location: difference Fourier map
*R*[*F*^2^ > 2σ(*F*^2^)] = 0.078	Hydrogen site location: inferred from neighbouring sites
*wR*(*F*^2^) = 0.242	H-atom parameters constrained
*S* = 1.03	*w* = 1/[σ^2^(*F*_o_^2^) + (0.1087*P*)^2^ + 0.1919*P*] where *P* = (*F*_o_^2^ + 2*F*_c_^2^)/3
12544 reflections	(Δ/σ)_max_ = 0.002
726 parameters	Δρ_max_ = 0.32 e Å^−^^3^
510 restraints	Δρ_min_ = −0.22 e Å^−^^3^

### Special details

**Table:**

Experimental. The crystal was placed in the cold stream of an Oxford Cryosystems Cobra open-flow nitrogen cryostat (Cosier & Glazer, 1986) operating at 100.0 (1) k.
Geometry. All s.u.'s (except the s.u. in the dihedral angle between two l.s. planes) are estimated using the full covariance matrix. The cell s.u.'s are taken into account individually in the estimation of s.u.'s in distances, angles and torsion angles; correlations between s.u.'s in cell parameters are only used when they are defined by crystal symmetry. An approximate (isotropic) treatment of cell s.u.'s is used for estimating s.u.'s involving l.s. planes.
Refinement. Refinement of F^2^ against ALL reflections. The weighted R-factor wR and goodness of fit S are based on F^2^, conventional R-factors R are based on F, with F set to zero for negative F^2^. The threshold expression of F^2^ > 2σ(F^2^) is used only for calculating R-factors(gt) etc. and is not relevant to the choice of reflections for refinement. R-factors based on F^2^ are statistically about twice as large as those based on F, and R- factors based on ALL data will be even larger.

### Fractional atomic coordinates and isotropic or equivalent isotropic displacement parameters (Å^2^)

**Table:**

	*x*	*y*	*z*	*U*_iso_*/*U*_eq_	Occ. (<1)
F2A	0.4613 (3)	0.09609 (11)	0.49187 (11)	0.1240 (6)	
O1A	0.9145 (2)	−0.05242 (8)	0.25336 (9)	0.0730 (5)	
N1A	0.9407 (2)	0.11515 (8)	0.17523 (8)	0.0456 (4)	
N2A	0.9414 (2)	0.06841 (9)	0.22753 (8)	0.0512 (4)	
F1A	1.1097 (13)	0.42500 (19)	0.0516 (2)	0.0741 (14)	0.72 (2)
C1A	1.1010 (14)	0.3317 (5)	0.2059 (4)	0.0436 (16)	0.72 (2)
H1AA	1.1266	0.3456	0.2583	0.052*	0.72 (2)
C2A	1.1175 (13)	0.3910 (4)	0.1671 (3)	0.0483 (15)	0.72 (2)
H2AA	1.1474	0.4451	0.1922	0.058*	0.72 (2)
C3A	1.0883 (11)	0.3676 (3)	0.0906 (3)	0.0477 (12)	0.72 (2)
C4A	1.0423 (10)	0.2887 (3)	0.0501 (3)	0.0489 (11)	0.72 (2)
H4AA	1.0252	0.2754	−0.0022	0.059*	0.72 (2)
C5A	1.0224 (9)	0.2300 (4)	0.0894 (4)	0.0431 (11)	0.72 (2)
H5AA	0.9927	0.1761	0.0636	0.052*	0.72 (2)
C6A	1.0466 (13)	0.2513 (4)	0.1680 (4)	0.0364 (12)	0.72 (2)
F1X	1.002 (4)	0.4294 (6)	0.0552 (7)	0.097 (5)	0.28 (2)
C1X	1.055 (3)	0.3297 (12)	0.2090 (11)	0.041 (4)	0.28 (2)
H1XA	1.0806	0.3444	0.2614	0.050*	0.28 (2)
C2X	1.062 (3)	0.3898 (12)	0.1703 (9)	0.052 (4)	0.28 (2)
H2XA	1.1014	0.4432	0.1964	0.062*	0.28 (2)
C3X	1.011 (3)	0.3712 (8)	0.0948 (8)	0.061 (4)	0.28 (2)
C4X	0.966 (4)	0.2924 (8)	0.0541 (9)	0.071 (4)	0.28 (2)
H4XA	0.9397	0.2791	0.0018	0.086*	0.28 (2)
C5X	0.962 (4)	0.2339 (10)	0.0943 (10)	0.066 (5)	0.28 (2)
H5XA	0.9226	0.1810	0.0671	0.079*	0.28 (2)
C6X	1.012 (4)	0.2471 (11)	0.1724 (11)	0.054 (5)	0.28 (2)
C7A	1.0168 (3)	0.18737 (10)	0.21017 (10)	0.0451 (4)	
C8A	1.0795 (3)	0.19916 (12)	0.29317 (11)	0.0615 (6)	
H8AA	1.0170	0.2399	0.3218	0.074*	
H8AB	1.2168	0.2143	0.3071	0.074*	
C9A	1.0154 (3)	0.11514 (11)	0.30573 (10)	0.0550 (5)	
H9AA	1.1276	0.0937	0.3258	0.066*	
C10A	0.8666 (3)	0.11110 (11)	0.35581 (10)	0.0523 (5)	
C11A	0.8752 (3)	0.06103 (12)	0.40491 (12)	0.0629 (6)	
H11A	0.9747	0.0307	0.4076	0.075*	
C12A	0.7375 (4)	0.05556 (14)	0.45006 (14)	0.0797 (7)	
H12A	0.7432	0.0215	0.4827	0.096*	
C13A	0.5947 (4)	0.10047 (15)	0.44597 (15)	0.0770 (7)	
C14A	0.5789 (3)	0.15065 (14)	0.39909 (13)	0.0708 (6)	
H14A	0.4783	0.1805	0.3973	0.085*	
C15A	0.7175 (3)	0.15619 (12)	0.35381 (11)	0.0613 (6)	
H15A	0.7102	0.1907	0.3217	0.074*	
C16A	0.8997 (3)	−0.01320 (11)	0.20599 (11)	0.0527 (5)	
C17A	0.8353 (3)	−0.04987 (11)	0.12432 (12)	0.0621 (6)	
H17A	0.8408	−0.1066	0.1142	0.093*	
H17B	0.9171	−0.0255	0.0960	0.093*	
H17C	0.7064	−0.0412	0.1100	0.093*	
F1B	0.6346 (2)	−0.09751 (7)	0.94366 (8)	0.0912 (5)	
F2B	1.2124 (3)	0.23406 (10)	0.50335 (11)	0.1207 (6)	
O1B	0.7863 (3)	0.38636 (8)	0.74826 (9)	0.0774 (5)	
N1B	0.7612 (2)	0.21708 (8)	0.82439 (8)	0.0474 (4)	
N2B	0.7549 (2)	0.26485 (9)	0.77306 (9)	0.0544 (4)	
C1B	0.5996 (3)	0.00176 (11)	0.79147 (11)	0.0512 (5)	
H1BA	0.5638	−0.0110	0.7394	0.061*	
C2B	0.5897 (3)	−0.05886 (11)	0.82958 (12)	0.0572 (5)	
H2BA	0.5488	−0.1122	0.8038	0.069*	
C3B	0.6414 (3)	−0.03835 (11)	0.90579 (12)	0.0575 (5)	
C4B	0.6973 (3)	0.03970 (11)	0.94695 (12)	0.0590 (5)	
H4BA	0.7271	0.0518	0.9992	0.071*	
C5B	0.7078 (3)	0.09941 (11)	0.90838 (11)	0.0516 (5)	
H5BA	0.7458	0.1526	0.9351	0.062*	
C6B	0.6628 (2)	0.08176 (10)	0.83014 (10)	0.0437 (4)	
C7B	0.6789 (3)	0.14553 (11)	0.78974 (10)	0.0458 (4)	
C8B	0.6035 (3)	0.13564 (12)	0.70780 (11)	0.0644 (6)	
H8BA	0.4653	0.1229	0.6973	0.077*	
H8BB	0.6576	0.0938	0.6765	0.077*	
C9B	0.6710 (3)	0.21961 (12)	0.69536 (11)	0.0564 (5)	
H9BA	0.5599	0.2430	0.6773	0.068*	
C10B	0.8155 (3)	0.22149 (11)	0.64289 (10)	0.0517 (5)	
C11B	0.8024 (3)	0.27024 (12)	0.59256 (11)	0.0616 (5)	
H11B	0.7022	0.3002	0.5900	0.074*	
C12B	0.9375 (4)	0.27470 (14)	0.54602 (13)	0.0791 (7)	
H12B	0.9295	0.3077	0.5125	0.095*	
C13B	1.0813 (4)	0.23014 (14)	0.55029 (14)	0.0749 (7)	
C14B	1.1007 (3)	0.18113 (13)	0.59823 (13)	0.0694 (6)	
H14B	1.2015	0.1514	0.5998	0.083*	
C15B	0.9645 (3)	0.17688 (12)	0.64496 (11)	0.0596 (5)	
H15B	0.9741	0.1435	0.6781	0.071*	
C16B	0.8064 (3)	0.34635 (11)	0.79500 (12)	0.0595 (5)	
C17B	0.8895 (4)	0.38121 (11)	0.87589 (12)	0.0752 (7)	
H17D	0.9314	0.4378	0.8837	0.113*	
H17E	0.9964	0.3551	0.8896	0.113*	
H17F	0.7944	0.3731	0.9065	0.113*	
F2C	0.1119 (3)	0.42973 (11)	0.48513 (12)	0.1249 (6)	
O1C	0.6321 (3)	0.28974 (9)	0.26097 (9)	0.0863 (6)	
N1C	0.6015 (2)	0.45434 (8)	0.18017 (8)	0.0458 (4)	
N2C	0.6263 (2)	0.40862 (9)	0.23277 (9)	0.0518 (4)	
F1C	0.6000 (10)	0.7682 (4)	0.0556 (5)	0.0767 (13)	0.67 (2)
C1C	0.712 (2)	0.6756 (5)	0.2086 (5)	0.0469 (16)	0.67 (2)
H1CA	0.7713	0.6896	0.2588	0.056*	0.67 (2)
C2C	0.6924 (16)	0.7348 (5)	0.1691 (4)	0.0506 (17)	0.67 (2)
H2CA	0.7318	0.7889	0.1926	0.061*	0.67 (2)
C3C	0.6135 (11)	0.7112 (4)	0.0953 (4)	0.0487 (13)	0.67 (2)
C4C	0.5451 (11)	0.6328 (3)	0.0575 (4)	0.0527 (12)	0.67 (2)
H4CA	0.4924	0.6196	0.0067	0.063*	0.67 (2)
C5C	0.5571 (12)	0.5745 (4)	0.0975 (4)	0.0461 (12)	0.67 (2)
H5CA	0.5068	0.5212	0.0741	0.055*	0.67 (2)
C6C	0.6446 (13)	0.5950 (5)	0.1731 (4)	0.0395 (14)	0.67 (2)
F1Y	0.671 (5)	0.7647 (9)	0.0556 (10)	0.123 (6)	0.33 (2)
C1Y	0.718 (5)	0.6637 (12)	0.2093 (10)	0.057 (4)	0.33 (2)
H1YA	0.7473	0.6776	0.2615	0.068*	0.33 (2)
C2Y	0.733 (3)	0.7245 (11)	0.1713 (9)	0.056 (4)	0.33 (2)
H2YA	0.7820	0.7771	0.1976	0.068*	0.33 (2)
C3Y	0.676 (3)	0.7066 (8)	0.0951 (9)	0.064 (4)	0.33 (2)
C4Y	0.623 (4)	0.6273 (7)	0.0549 (8)	0.075 (4)	0.33 (2)
H4YA	0.5864	0.6145	0.0029	0.090*	0.33 (2)
C5Y	0.626 (3)	0.5680 (8)	0.0937 (8)	0.056 (3)	0.33 (2)
H5YA	0.6051	0.5148	0.0660	0.068*	0.33 (2)
C6Y	0.660 (3)	0.5826 (11)	0.1730 (8)	0.057 (4)	0.33 (2)
C7C	0.6597 (2)	0.52828 (10)	0.21396 (10)	0.0450 (4)	
C8C	0.7417 (3)	0.54173 (12)	0.29583 (11)	0.0606 (5)	
H8CA	0.6840	0.5823	0.3270	0.073*	
H8CB	0.8791	0.5577	0.3049	0.073*	
C9C	0.6887 (3)	0.45806 (11)	0.31055 (11)	0.0552 (5)	
H9CA	0.8033	0.4405	0.3337	0.066*	
C10C	0.5332 (3)	0.45217 (11)	0.35786 (10)	0.0506 (5)	
C11C	0.5377 (3)	0.40019 (12)	0.40562 (11)	0.0615 (5)	
H11C	0.6378	0.3703	0.4090	0.074*	
C12C	0.3949 (4)	0.39252 (14)	0.44809 (14)	0.0780 (7)	
H12C	0.3976	0.3577	0.4799	0.094*	
C13C	0.2514 (4)	0.43678 (15)	0.44227 (15)	0.0762 (7)	
C14C	0.2400 (3)	0.48843 (13)	0.39656 (13)	0.0682 (6)	
H14C	0.1389	0.5179	0.3939	0.082*	
C15C	0.3827 (3)	0.49603 (12)	0.35406 (11)	0.0572 (5)	
H15C	0.3775	0.5311	0.3225	0.069*	
C16C	0.6052 (3)	0.32713 (11)	0.21266 (12)	0.0598 (5)	
C17C	0.5497 (4)	0.28828 (11)	0.13118 (12)	0.0732 (7)	
H17G	0.5667	0.2327	0.1224	0.110*	
H17H	0.6285	0.3149	0.1033	0.110*	
H17I	0.4182	0.2921	0.1151	0.110*	

### Atomic displacement parameters (Å^2^)

**Table:**

	*U*^11^	*U*^22^	*U*^33^	*U*^12^	*U*^13^	*U*^23^
F2A	0.1377 (15)	0.1098 (12)	0.1409 (15)	0.0033 (11)	0.0792 (13)	0.0377 (11)
O1A	0.1061 (12)	0.0531 (8)	0.0716 (10)	0.0171 (8)	0.0127 (9)	0.0381 (8)
N1A	0.0561 (9)	0.0378 (8)	0.0454 (9)	0.0016 (6)	0.0047 (7)	0.0202 (7)
N2A	0.0690 (10)	0.0413 (8)	0.0442 (9)	−0.0003 (7)	0.0014 (7)	0.0215 (7)
F1A	0.123 (4)	0.0429 (10)	0.0600 (13)	−0.0076 (16)	0.0184 (17)	0.0272 (9)
C1A	0.044 (4)	0.042 (2)	0.039 (2)	−0.0108 (19)	0.005 (2)	0.0067 (15)
C2A	0.058 (4)	0.0319 (16)	0.051 (2)	−0.010 (2)	0.0107 (19)	0.0091 (14)
C3A	0.060 (3)	0.0363 (15)	0.0505 (18)	−0.0030 (17)	0.0139 (19)	0.0195 (13)
C4A	0.066 (3)	0.0417 (16)	0.0386 (17)	−0.0042 (17)	0.0114 (18)	0.0132 (13)
C5A	0.055 (3)	0.0323 (15)	0.0397 (18)	−0.0034 (15)	0.0084 (17)	0.0088 (13)
C6A	0.034 (2)	0.0368 (19)	0.040 (2)	−0.0050 (15)	0.0039 (14)	0.0207 (17)
F1X	0.151 (13)	0.049 (3)	0.090 (5)	−0.012 (5)	0.008 (6)	0.037 (3)
C1X	0.041 (9)	0.035 (4)	0.051 (5)	−0.006 (4)	0.024 (5)	0.014 (3)
C2X	0.056 (9)	0.037 (4)	0.059 (5)	−0.011 (5)	0.020 (5)	0.008 (4)
C3X	0.073 (9)	0.043 (4)	0.065 (5)	−0.013 (5)	0.012 (6)	0.022 (4)
C4X	0.104 (12)	0.051 (5)	0.056 (5)	−0.017 (7)	0.016 (7)	0.019 (4)
C5X	0.099 (12)	0.039 (5)	0.055 (5)	−0.012 (7)	0.016 (7)	0.013 (4)
C6X	0.064 (11)	0.041 (5)	0.044 (5)	−0.018 (5)	0.007 (6)	−0.003 (5)
C7A	0.0499 (10)	0.0398 (9)	0.0447 (11)	−0.0031 (8)	0.0025 (8)	0.0165 (8)
C8A	0.0758 (14)	0.0540 (12)	0.0491 (12)	−0.0158 (10)	−0.0053 (10)	0.0227 (10)
C9A	0.0645 (12)	0.0543 (11)	0.0447 (11)	−0.0010 (9)	−0.0061 (9)	0.0230 (9)
C10A	0.0677 (13)	0.0455 (10)	0.0398 (10)	−0.0049 (9)	−0.0070 (9)	0.0182 (9)
C11A	0.0840 (15)	0.0545 (12)	0.0549 (13)	0.0068 (10)	0.0051 (11)	0.0287 (10)
C12A	0.117 (2)	0.0615 (14)	0.0678 (16)	−0.0007 (14)	0.0212 (15)	0.0347 (12)
C13A	0.0909 (18)	0.0617 (14)	0.0769 (17)	−0.0080 (13)	0.0264 (14)	0.0155 (13)
C14A	0.0716 (15)	0.0642 (14)	0.0698 (15)	0.0057 (11)	0.0023 (12)	0.0099 (12)
C15A	0.0786 (15)	0.0510 (11)	0.0498 (12)	0.0030 (10)	−0.0097 (11)	0.0187 (10)
C16A	0.0621 (12)	0.0418 (10)	0.0611 (13)	0.0092 (8)	0.0108 (10)	0.0258 (10)
C17A	0.0827 (15)	0.0387 (10)	0.0664 (14)	0.0049 (9)	0.0156 (11)	0.0160 (10)
F1B	0.1521 (14)	0.0463 (7)	0.0769 (9)	−0.0046 (7)	0.0106 (9)	0.0329 (7)
F2B	0.1402 (15)	0.1065 (12)	0.1281 (14)	−0.0002 (10)	0.0764 (12)	0.0311 (11)
O1B	0.1182 (13)	0.0545 (8)	0.0744 (10)	0.0239 (8)	0.0208 (9)	0.0383 (8)
N1B	0.0634 (10)	0.0389 (8)	0.0458 (9)	0.0084 (7)	0.0122 (7)	0.0200 (7)
N2B	0.0790 (11)	0.0437 (9)	0.0444 (9)	0.0061 (8)	0.0083 (8)	0.0216 (7)
C1B	0.0632 (12)	0.0436 (10)	0.0438 (11)	−0.0027 (8)	0.0104 (9)	0.0083 (9)
C2B	0.0758 (14)	0.0340 (9)	0.0596 (13)	−0.0017 (9)	0.0122 (10)	0.0108 (9)
C3B	0.0749 (14)	0.0400 (10)	0.0610 (14)	0.0002 (9)	0.0114 (11)	0.0235 (10)
C4B	0.0797 (14)	0.0458 (11)	0.0491 (12)	−0.0032 (9)	0.0018 (10)	0.0181 (9)
C5B	0.0676 (12)	0.0363 (9)	0.0467 (11)	−0.0037 (8)	0.0010 (9)	0.0119 (8)
C6B	0.0463 (10)	0.0387 (9)	0.0468 (11)	0.0009 (7)	0.0074 (8)	0.0146 (8)
C7B	0.0499 (10)	0.0434 (10)	0.0445 (11)	0.0005 (8)	0.0066 (8)	0.0157 (8)
C8B	0.0770 (14)	0.0614 (13)	0.0510 (12)	−0.0118 (10)	−0.0034 (10)	0.0255 (10)
C9B	0.0654 (13)	0.0566 (11)	0.0477 (12)	0.0016 (9)	−0.0025 (9)	0.0247 (10)
C10B	0.0679 (12)	0.0462 (10)	0.0371 (10)	−0.0036 (9)	−0.0063 (9)	0.0170 (8)
C11B	0.0828 (15)	0.0537 (12)	0.0518 (12)	0.0065 (10)	0.0032 (11)	0.0271 (10)
C12B	0.119 (2)	0.0616 (14)	0.0610 (15)	−0.0049 (14)	0.0171 (14)	0.0315 (12)
C13B	0.0900 (18)	0.0596 (14)	0.0729 (16)	−0.0089 (12)	0.0261 (14)	0.0135 (12)
C14B	0.0735 (15)	0.0636 (14)	0.0647 (15)	0.0058 (11)	0.0044 (12)	0.0089 (12)
C15B	0.0762 (14)	0.0528 (11)	0.0470 (12)	0.0067 (10)	−0.0060 (10)	0.0178 (9)
C16B	0.0845 (15)	0.0431 (11)	0.0626 (14)	0.0166 (10)	0.0238 (11)	0.0265 (10)
C17B	0.130 (2)	0.0381 (10)	0.0620 (14)	0.0134 (12)	0.0289 (14)	0.0122 (10)
F2C	0.1355 (15)	0.1059 (12)	0.1501 (16)	0.0022 (10)	0.0853 (13)	0.0360 (11)
O1C	0.1375 (16)	0.0627 (9)	0.0793 (11)	0.0351 (10)	0.0268 (10)	0.0446 (9)
N1C	0.0557 (9)	0.0376 (8)	0.0477 (9)	0.0036 (6)	0.0081 (7)	0.0195 (7)
N2C	0.0712 (11)	0.0433 (8)	0.0447 (9)	0.0048 (7)	0.0063 (8)	0.0222 (7)
F1C	0.117 (3)	0.0422 (15)	0.081 (2)	0.0150 (16)	0.021 (2)	0.0325 (15)
C1C	0.059 (3)	0.030 (3)	0.046 (3)	−0.006 (2)	0.006 (2)	0.005 (2)
C2C	0.072 (4)	0.025 (2)	0.060 (3)	0.007 (2)	0.022 (2)	0.0142 (17)
C3C	0.059 (3)	0.0343 (18)	0.060 (2)	0.0096 (17)	0.020 (2)	0.0203 (16)
C4C	0.072 (3)	0.0406 (18)	0.047 (2)	0.011 (2)	0.006 (2)	0.0161 (15)
C5C	0.059 (3)	0.0294 (17)	0.0475 (19)	0.0037 (18)	0.004 (2)	0.0092 (14)
C6C	0.055 (3)	0.025 (2)	0.044 (2)	0.0003 (18)	0.018 (2)	0.0181 (19)
F1Y	0.238 (17)	0.042 (4)	0.075 (6)	−0.013 (7)	−0.019 (9)	0.031 (4)
C1Y	0.093 (9)	0.035 (6)	0.050 (6)	−0.001 (6)	0.027 (6)	0.023 (4)
C2Y	0.078 (9)	0.023 (5)	0.059 (5)	0.007 (5)	−0.004 (5)	0.001 (4)
C3Y	0.098 (11)	0.035 (4)	0.058 (5)	−0.002 (5)	0.003 (6)	0.020 (4)
C4Y	0.119 (11)	0.036 (4)	0.054 (5)	−0.002 (6)	−0.022 (7)	0.007 (3)
C5Y	0.081 (9)	0.033 (4)	0.051 (4)	0.001 (5)	−0.007 (5)	0.015 (3)
C6Y	0.073 (7)	0.030 (5)	0.053 (5)	−0.002 (5)	−0.019 (5)	−0.003 (4)
C7C	0.0490 (10)	0.0410 (10)	0.0445 (11)	−0.0046 (8)	0.0043 (8)	0.0166 (8)
C8C	0.0690 (13)	0.0567 (12)	0.0504 (12)	−0.0149 (9)	−0.0031 (10)	0.0211 (10)
C9C	0.0603 (12)	0.0562 (12)	0.0493 (12)	−0.0026 (9)	−0.0034 (9)	0.0260 (10)
C10C	0.0608 (12)	0.0488 (10)	0.0387 (10)	−0.0041 (9)	−0.0053 (8)	0.0174 (8)
C11C	0.0801 (15)	0.0575 (12)	0.0516 (12)	0.0080 (10)	0.0051 (11)	0.0277 (10)
C12C	0.111 (2)	0.0615 (14)	0.0675 (16)	−0.0037 (14)	0.0241 (14)	0.0316 (12)
C13C	0.0844 (17)	0.0630 (14)	0.0797 (17)	−0.0094 (12)	0.0307 (14)	0.0134 (13)
C14C	0.0655 (14)	0.0614 (13)	0.0707 (15)	0.0018 (10)	0.0034 (12)	0.0099 (12)
C15C	0.0665 (13)	0.0533 (11)	0.0484 (12)	0.0012 (9)	−0.0063 (10)	0.0187 (9)
C16C	0.0784 (14)	0.0439 (11)	0.0679 (14)	0.0164 (10)	0.0197 (11)	0.0281 (10)
C17C	0.1102 (19)	0.0383 (10)	0.0728 (16)	0.0099 (11)	0.0195 (14)	0.0147 (11)

### Geometric parameters (Å, °)

**Table:**

F2A—C13A	1.368 (3)	C8B—C9B	1.550 (3)
O1A—C16A	1.220 (2)	C8B—H8BA	0.97
N1A—C7A	1.287 (2)	C8B—H8BB	0.97
N1A—N2A	1.3895 (18)	C9B—C10B	1.514 (3)
N2A—C16A	1.360 (2)	C9B—H9BA	0.98
N2A—C9A	1.481 (2)	C10B—C15B	1.382 (3)
F1A—C3A	1.355 (4)	C10B—C11B	1.386 (2)
C1A—C2A	1.379 (6)	C11B—C12B	1.386 (3)
C1A—C6A	1.390 (6)	C11B—H11B	0.93
C1A—H1AA	0.93	C12B—C13B	1.353 (4)
C2A—C3A	1.360 (5)	C12B—H12B	0.93
C2A—H2AA	0.93	C13B—C14B	1.358 (3)
C3A—C4A	1.377 (5)	C14B—C15B	1.394 (3)
C4A—C5A	1.376 (5)	C14B—H14B	0.93
C4A—H4AA	0.93	C15B—H15B	0.93
C5A—C6A	1.399 (6)	C16B—C17B	1.492 (3)
C5A—H5AA	0.93	C17B—H17D	0.96
C6A—C7A	1.497 (6)	C17B—H17E	0.96
F1X—C3X	1.369 (11)	C17B—H17F	0.96
C1X—C2X	1.385 (13)	F2C—C13C	1.368 (3)
C1X—C6X	1.413 (13)	O1C—C16C	1.219 (2)
C1X—H1XA	0.93	N1C—C7C	1.282 (2)
C2X—C3X	1.348 (13)	N1C—N2C	1.3867 (18)
C2X—H2XA	0.93	N2C—C16C	1.356 (2)
C3X—C4X	1.377 (12)	N2C—C9C	1.478 (2)
C4X—C5X	1.379 (13)	F1C—C3C	1.355 (6)
C4X—H4XA	0.93	C1C—C2C	1.390 (7)
C5X—C6X	1.399 (14)	C1C—C6C	1.396 (6)
C5X—H5XA	0.93	C1C—H1CA	0.93
C6X—C7A	1.367 (18)	C2C—C3C	1.354 (7)
C7A—C8A	1.499 (2)	C2C—H2CA	0.93
C8A—C9A	1.547 (2)	C3C—C4C	1.375 (6)
C8A—H8AA	0.97	C4C—C5C	1.376 (6)
C8A—H8AB	0.97	C4C—H4CA	0.93
C9A—C10A	1.509 (3)	C5C—C6C	1.400 (7)
C9A—H9AA	0.98	C5C—H5CA	0.93
C10A—C11A	1.385 (2)	C6C—C7C	1.516 (7)
C10A—C15A	1.388 (3)	F1Y—C3Y	1.365 (11)
C11A—C12A	1.384 (3)	C1Y—C2Y	1.388 (13)
C11A—H11A	0.93	C1Y—C6Y	1.396 (12)
C12A—C13A	1.351 (4)	C1Y—H1YA	0.93
C12A—H12A	0.93	C2Y—C3Y	1.367 (12)
C13A—C14A	1.357 (3)	C2Y—H2YA	0.93
C14A—C15A	1.391 (3)	C3Y—C4Y	1.384 (11)
C14A—H14A	0.93	C4Y—C5Y	1.373 (11)
C15A—H15A	0.93	C4Y—H4YA	0.93
C16A—C17A	1.486 (3)	C5Y—C6Y	1.412 (12)
C17A—H17A	0.96	C5Y—H5YA	0.93
C17A—H17B	0.96	C6Y—C7C	1.329 (16)
C17A—H17C	0.96	C7C—C8C	1.502 (2)
F1B—C3B	1.360 (2)	C8C—C9C	1.542 (2)
F2B—C13B	1.368 (3)	C8C—H8CA	0.97
O1B—C16B	1.223 (2)	C8C—H8CB	0.97
N1B—C7B	1.288 (2)	C9C—C10C	1.514 (3)
N1B—N2B	1.3873 (18)	C9C—H9CA	0.98
N2B—C16B	1.365 (2)	C10C—C15C	1.384 (3)
N2B—C9B	1.480 (2)	C10C—C11C	1.391 (2)
C1B—C2B	1.385 (2)	C11C—C12C	1.383 (3)
C1B—C6B	1.396 (2)	C11C—H11C	0.93
C1B—H1BA	0.93	C12C—C13C	1.351 (4)
C2B—C3B	1.361 (3)	C12C—H12C	0.93
C2B—H2BA	0.93	C13C—C14C	1.358 (3)
C3B—C4B	1.374 (3)	C14C—C15C	1.383 (3)
C4B—C5B	1.377 (2)	C14C—H14C	0.93
C4B—H4BA	0.93	C15C—H15C	0.93
C5B—C6B	1.394 (3)	C16C—C17C	1.483 (3)
C5B—H5BA	0.93	C17C—H17G	0.96
C6B—C7B	1.463 (2)	C17C—H17H	0.96
C7B—C8B	1.501 (3)	C17C—H17I	0.96
			
C7A—N1A—N2A	108.01 (14)	N2B—C9B—C8B	101.28 (14)
C16A—N2A—N1A	121.67 (15)	C10B—C9B—C8B	115.55 (17)
C16A—N2A—C9A	124.59 (15)	N2B—C9B—H9BA	109.5
N1A—N2A—C9A	113.27 (13)	C10B—C9B—H9BA	109.5
C2A—C1A—C6A	121.0 (5)	C8B—C9B—H9BA	109.5
C2A—C1A—H1AA	119.5	C15B—C10B—C11B	118.6 (2)
C6A—C1A—H1AA	119.5	C15B—C10B—C9B	121.48 (16)
C3A—C2A—C1A	117.5 (5)	C11B—C10B—C9B	119.88 (18)
C3A—C2A—H2AA	121.2	C12B—C11B—C10B	120.5 (2)
C1A—C2A—H2AA	121.2	C12B—C11B—H11B	119.7
F1A—C3A—C2A	118.2 (4)	C10B—C11B—H11B	119.7
F1A—C3A—C4A	117.7 (4)	C13B—C12B—C11B	118.7 (2)
C2A—C3A—C4A	124.1 (4)	C13B—C12B—H12B	120.6
C5A—C4A—C3A	117.9 (5)	C11B—C12B—H12B	120.6
C5A—C4A—H4AA	121.0	C12B—C13B—C14B	123.3 (2)
C3A—C4A—H4AA	121.0	C12B—C13B—F2B	118.3 (2)
C4A—C5A—C6A	120.1 (5)	C14B—C13B—F2B	118.4 (2)
C4A—C5A—H5AA	119.9	C13B—C14B—C15B	117.8 (2)
C6A—C5A—H5AA	119.9	C13B—C14B—H14B	121.1
C1A—C6A—C5A	119.2 (5)	C15B—C14B—H14B	121.1
C1A—C6A—C7A	121.0 (5)	C10B—C15B—C14B	121.08 (19)
C5A—C6A—C7A	119.8 (5)	C10B—C15B—H15B	119.5
C2X—C1X—C6X	122.8 (15)	C14B—C15B—H15B	119.5
C2X—C1X—H1XA	118.6	O1B—C16B—N2B	119.4 (2)
C6X—C1X—H1XA	118.6	O1B—C16B—C17B	123.72 (18)
C3X—C2X—C1X	120.2 (15)	N2B—C16B—C17B	116.87 (16)
C3X—C2X—H2XA	119.9	C16B—C17B—H17D	109.5
C1X—C2X—H2XA	119.9	C16B—C17B—H17E	109.5
C2X—C3X—F1X	121.8 (12)	H17D—C17B—H17E	109.5
C2X—C3X—C4X	121.1 (12)	C16B—C17B—H17F	109.5
F1X—C3X—C4X	117.1 (12)	H17D—C17B—H17F	109.5
C3X—C4X—C5X	117.1 (13)	H17E—C17B—H17F	109.5
C3X—C4X—H4XA	121.4	C7C—N1C—N2C	108.00 (14)
C5X—C4X—H4XA	121.4	C16C—N2C—N1C	122.17 (16)
C4X—C5X—C6X	125.9 (14)	C16C—N2C—C9C	124.71 (15)
C4X—C5X—H5XA	117.1	N1C—N2C—C9C	113.01 (13)
C6X—C5X—H5XA	117.1	C2C—C1C—C6C	120.1 (6)
C7A—C6X—C5X	124.4 (15)	C2C—C1C—H1CA	119.9
C7A—C6X—C1X	123.0 (14)	C6C—C1C—H1CA	119.9
C5X—C6X—C1X	112.6 (14)	C3C—C2C—C1C	117.9 (6)
N1A—C7A—C6X	119.1 (8)	C3C—C2C—H2CA	121.1
N1A—C7A—C6A	120.7 (3)	C1C—C2C—H2CA	121.1
N1A—C7A—C8A	114.60 (15)	C2C—C3C—F1C	118.3 (6)
C6X—C7A—C8A	125.8 (8)	C2C—C3C—C4C	124.4 (6)
C6A—C7A—C8A	124.6 (3)	F1C—C3C—C4C	117.4 (6)
C7A—C8A—C9A	102.63 (15)	C3C—C4C—C5C	117.8 (6)
C7A—C8A—H8AA	111.2	C3C—C4C—H4CA	121.1
C9A—C8A—H8AA	111.2	C5C—C4C—H4CA	121.1
C7A—C8A—H8AB	111.2	C4C—C5C—C6C	120.3 (5)
C9A—C8A—H8AB	111.2	C4C—C5C—H5CA	119.9
H8AA—C8A—H8AB	109.2	C6C—C5C—H5CA	119.9
N2A—C9A—C10A	111.54 (15)	C1C—C6C—C5C	119.5 (6)
N2A—C9A—C8A	101.25 (13)	C1C—C6C—C7C	122.2 (6)
C10A—C9A—C8A	116.02 (17)	C5C—C6C—C7C	118.2 (5)
N2A—C9A—H9AA	109.2	C2Y—C1Y—C6Y	123.4 (14)
C10A—C9A—H9AA	109.2	C2Y—C1Y—H1YA	118.3
C8A—C9A—H9AA	109.2	C6Y—C1Y—H1YA	118.3
C11A—C10A—C15A	118.2 (2)	C3Y—C2Y—C1Y	119.5 (13)
C11A—C10A—C9A	120.21 (18)	C3Y—C2Y—H2YA	120.2
C15A—C10A—C9A	121.60 (17)	C1Y—C2Y—H2YA	120.2
C12A—C11A—C10A	120.8 (2)	F1Y—C3Y—C2Y	122.2 (13)
C12A—C11A—H11A	119.6	F1Y—C3Y—C4Y	117.5 (12)
C10A—C11A—H11A	119.6	C2Y—C3Y—C4Y	120.2 (11)
C13A—C12A—C11A	118.9 (2)	C5Y—C4Y—C3Y	118.4 (11)
C13A—C12A—H12A	120.6	C5Y—C4Y—H4YA	120.8
C11A—C12A—H12A	120.6	C3Y—C4Y—H4YA	120.8
C12A—C13A—C14A	123.0 (2)	C4Y—C5Y—C6Y	124.3 (12)
C12A—C13A—F2A	118.6 (2)	C4Y—C5Y—H5YA	117.9
C14A—C13A—F2A	118.4 (3)	C6Y—C5Y—H5YA	117.9
C13A—C14A—C15A	118.1 (2)	C7C—C6Y—C1Y	118.9 (13)
C13A—C14A—H14A	121.0	C7C—C6Y—C5Y	127.3 (13)
C15A—C14A—H14A	121.0	C1Y—C6Y—C5Y	113.4 (13)
C10A—C15A—C14A	121.08 (19)	N1C—C7C—C6Y	118.2 (7)
C10A—C15A—H15A	119.5	N1C—C7C—C8C	114.27 (15)
C14A—C15A—H15A	119.5	C6Y—C7C—C8C	127.2 (7)
O1A—C16A—N2A	119.64 (18)	N1C—C7C—C6C	121.6 (3)
O1A—C16A—C17A	123.35 (17)	C8C—C7C—C6C	124.1 (3)
N2A—C16A—C17A	117.00 (15)	C7C—C8C—C9C	102.18 (15)
C16A—C17A—H17A	109.5	C7C—C8C—H8CA	111.3
C16A—C17A—H17B	109.5	C9C—C8C—H8CA	111.3
H17A—C17A—H17B	109.5	C7C—C8C—H8CB	111.3
C16A—C17A—H17C	109.5	C9C—C8C—H8CB	111.3
H17A—C17A—H17C	109.5	H8CA—C8C—H8CB	109.2
H17B—C17A—H17C	109.5	N2C—C9C—C10C	111.47 (15)
C7B—N1B—N2B	108.18 (15)	N2C—C9C—C8C	100.87 (13)
C16B—N2B—N1B	121.94 (16)	C10C—C9C—C8C	115.30 (17)
C16B—N2B—C9B	124.58 (15)	N2C—C9C—H9CA	109.6
N1B—N2B—C9B	113.25 (14)	C10C—C9C—H9CA	109.6
C2B—C1B—C6B	120.88 (18)	C8C—C9C—H9CA	109.6
C2B—C1B—H1BA	119.6	C15C—C10C—C11C	118.38 (19)
C6B—C1B—H1BA	119.6	C15C—C10C—C9C	121.91 (16)
C3B—C2B—C1B	118.29 (17)	C11C—C10C—C9C	119.70 (18)
C3B—C2B—H2BA	120.9	C12C—C11C—C10C	120.7 (2)
C1B—C2B—H2BA	120.9	C12C—C11C—H11C	119.7
F1B—C3B—C2B	118.77 (17)	C10C—C11C—H11C	119.7
F1B—C3B—C4B	117.96 (18)	C13C—C12C—C11C	118.5 (2)
C2B—C3B—C4B	123.26 (17)	C13C—C12C—H12C	120.7
C3B—C4B—C5B	117.89 (18)	C11C—C12C—H12C	120.7
C3B—C4B—H4BA	121.1	C12C—C13C—C14C	123.2 (2)
C5B—C4B—H4BA	121.1	C12C—C13C—F2C	118.2 (2)
C4B—C5B—C6B	121.40 (17)	C14C—C13C—F2C	118.5 (2)
C4B—C5B—H5BA	119.3	C13C—C14C—C15C	118.2 (2)
C6B—C5B—H5BA	119.3	C13C—C14C—H14C	120.9
C5B—C6B—C1B	118.20 (16)	C15C—C14C—H14C	120.9
C5B—C6B—C7B	120.86 (16)	C14C—C15C—C10C	120.96 (19)
C1B—C6B—C7B	120.94 (17)	C14C—C15C—H15C	119.5
N1B—C7B—C6B	120.47 (16)	C10C—C15C—H15C	119.5
N1B—C7B—C8B	114.47 (15)	O1C—C16C—N2C	119.7 (2)
C6B—C7B—C8B	125.06 (16)	O1C—C16C—C17C	123.49 (18)
C7B—C8B—C9B	102.45 (15)	N2C—C16C—C17C	116.85 (16)
C7B—C8B—H8BA	111.3	C16C—C17C—H17G	109.5
C9B—C8B—H8BA	111.3	C16C—C17C—H17H	109.5
C7B—C8B—H8BB	111.3	H17G—C17C—H17H	109.5
C9B—C8B—H8BB	111.3	C16C—C17C—H17I	109.5
H8BA—C8B—H8BB	109.2	H17G—C17C—H17I	109.5
N2B—C9B—C10B	111.14 (16)	H17H—C17C—H17I	109.5
			
C7A—N1A—N2A—C16A	−168.87 (17)	C16B—N2B—C9B—C8B	−168.37 (19)
C7A—N1A—N2A—C9A	3.5 (2)	N1B—N2B—C9B—C8B	6.1 (2)
C6A—C1A—C2A—C3A	−3.3 (11)	C7B—C8B—C9B—N2B	−5.5 (2)
C1A—C2A—C3A—F1A	−178.0 (6)	C7B—C8B—C9B—C10B	114.70 (18)
C1A—C2A—C3A—C4A	0.5 (9)	N2B—C9B—C10B—C15B	73.5 (2)
F1A—C3A—C4A—C5A	179.3 (4)	C8B—C9B—C10B—C15B	−41.2 (2)
C2A—C3A—C4A—C5A	0.8 (7)	N2B—C9B—C10B—C11B	−104.97 (19)
C3A—C4A—C5A—C6A	0.7 (7)	C8B—C9B—C10B—C11B	140.37 (18)
C2A—C1A—C6A—C5A	4.8 (12)	C15B—C10B—C11B—C12B	−0.8 (3)
C2A—C1A—C6A—C7A	−176.1 (7)	C9B—C10B—C11B—C12B	177.75 (19)
C4A—C5A—C6A—C1A	−3.4 (11)	C10B—C11B—C12B—C13B	0.5 (3)
C4A—C5A—C6A—C7A	177.5 (5)	C11B—C12B—C13B—C14B	−0.2 (4)
C6X—C1X—C2X—C3X	5(3)	C11B—C12B—C13B—F2B	179.1 (2)
C1X—C2X—C3X—F1X	175.1 (15)	C12B—C13B—C14B—C15B	0.2 (4)
C1X—C2X—C3X—C4X	−5(2)	F2B—C13B—C14B—C15B	−179.1 (2)
C2X—C3X—C4X—C5X	5(2)	C11B—C10B—C15B—C14B	0.7 (3)
F1X—C3X—C4X—C5X	−175.6 (12)	C9B—C10B—C15B—C14B	−177.75 (18)
C3X—C4X—C5X—C6X	−5(2)	C13B—C14B—C15B—C10B	−0.4 (3)
C4X—C5X—C6X—C7A	−176.1 (18)	N1B—N2B—C16B—O1B	−175.16 (17)
C4X—C5X—C6X—C1X	5(3)	C9B—N2B—C16B—O1B	−1.1 (3)
C2X—C1X—C6X—C7A	176 (2)	N1B—N2B—C16B—C17B	6.2 (3)
C2X—C1X—C6X—C5X	−5(3)	C9B—N2B—C16B—C17B	−179.76 (19)
N2A—N1A—C7A—C6X	−172.3 (15)	C7C—N1C—N2C—C16C	−169.85 (17)
N2A—N1A—C7A—C6A	176.6 (4)	C7C—N1C—N2C—C9C	6.5 (2)
N2A—N1A—C7A—C8A	−0.3 (2)	C6C—C1C—C2C—C3C	−2.9 (18)
C5X—C6X—C7A—N1A	−12 (3)	C1C—C2C—C3C—F1C	−177.5 (10)
C1X—C6X—C7A—N1A	166.6 (18)	C1C—C2C—C3C—C4C	2.7 (14)
C5X—C6X—C7A—C6A	90 (7)	C2C—C3C—C4C—C5C	0.2 (9)
C1X—C6X—C7A—C6A	−91 (7)	F1C—C3C—C4C—C5C	−179.6 (5)
C5X—C6X—C7A—C8A	176.6 (16)	C3C—C4C—C5C—C6C	−2.9 (8)
C1X—C6X—C7A—C8A	−4(3)	C2C—C1C—C6C—C5C	0.3 (18)
C1A—C6A—C7A—N1A	170.0 (6)	C2C—C1C—C6C—C7C	−178.9 (10)
C5A—C6A—C7A—N1A	−10.9 (9)	C4C—C5C—C6C—C1C	2.7 (12)
C1A—C6A—C7A—C6X	87 (6)	C4C—C5C—C6C—C7C	−178.1 (5)
C5A—C6A—C7A—C6X	−94 (6)	C6Y—C1Y—C2Y—C3Y	5(4)
C1A—C6A—C7A—C8A	−13.4 (10)	C1Y—C2Y—C3Y—F1Y	174 (2)
C5A—C6A—C7A—C8A	165.7 (5)	C1Y—C2Y—C3Y—C4Y	−7(3)
N1A—C7A—C8A—C9A	−2.7 (2)	F1Y—C3Y—C4Y—C5Y	−179.6 (12)
C6X—C7A—C8A—C9A	168.6 (16)	C2Y—C3Y—C4Y—C5Y	1(2)
C6A—C7A—C8A—C9A	−179.4 (5)	C3Y—C4Y—C5Y—C6Y	7(2)
C16A—N2A—C9A—C10A	−68.8 (2)	C2Y—C1Y—C6Y—C7C	176 (3)
N1A—N2A—C9A—C10A	119.06 (16)	C2Y—C1Y—C6Y—C5Y	3(4)
C16A—N2A—C9A—C8A	167.22 (18)	C4Y—C5Y—C6Y—C7C	178.1 (15)
N1A—N2A—C9A—C8A	−4.9 (2)	C4Y—C5Y—C6Y—C1Y	−9(3)
C7A—C8A—C9A—N2A	4.23 (19)	N2C—N1C—C7C—C6Y	176.0 (12)
C7A—C8A—C9A—C10A	−116.68 (18)	N2C—N1C—C7C—C8C	2.6 (2)
N2A—C9A—C10A—C11A	102.35 (19)	N2C—N1C—C7C—C6C	−177.3 (4)
C8A—C9A—C10A—C11A	−142.43 (18)	C1Y—C6Y—C7C—N1C	178 (2)
N2A—C9A—C10A—C15A	−77.0 (2)	C5Y—C6Y—C7C—N1C	−9(3)
C8A—C9A—C10A—C15A	38.2 (2)	C1Y—C6Y—C7C—C8C	−9(3)
C15A—C10A—C11A—C12A	0.7 (3)	C5Y—C6Y—C7C—C8C	163.3 (15)
C9A—C10A—C11A—C12A	−178.70 (19)	C1Y—C6Y—C7C—C6C	56 (8)
C10A—C11A—C12A—C13A	−0.5 (4)	C5Y—C6Y—C7C—C6C	−131 (10)
C11A—C12A—C13A—C14A	0.4 (4)	C1C—C6C—C7C—N1C	−178.1 (9)
C11A—C12A—C13A—F2A	−178.6 (2)	C5C—C6C—C7C—N1C	2.8 (9)
C12A—C13A—C14A—C15A	−0.5 (4)	C1C—C6C—C7C—C6Y	−117 (9)
F2A—C13A—C14A—C15A	178.6 (2)	C5C—C6C—C7C—C6Y	64 (8)
C11A—C10A—C15A—C14A	−0.8 (3)	C1C—C6C—C7C—C8C	2.0 (12)
C9A—C10A—C15A—C14A	178.59 (18)	C5C—C6C—C7C—C8C	−177.1 (5)
C13A—C14A—C15A—C10A	0.7 (3)	N1C—C7C—C8C—C9C	−9.8 (2)
N1A—N2A—C16A—O1A	175.02 (16)	C6Y—C7C—C8C—C9C	177.4 (13)
C9A—N2A—C16A—O1A	3.5 (3)	C6C—C7C—C8C—C9C	170.1 (4)
N1A—N2A—C16A—C17A	−5.7 (3)	C16C—N2C—C9C—C10C	−72.8 (2)
C9A—N2A—C16A—C17A	−177.21 (18)	N1C—N2C—C9C—C10C	110.90 (16)
C7B—N1B—N2B—C16B	170.59 (18)	C16C—N2C—C9C—C8C	164.28 (18)
C7B—N1B—N2B—C9B	−4.1 (2)	N1C—N2C—C9C—C8C	−12.0 (2)
C6B—C1B—C2B—C3B	0.6 (3)	C7C—C8C—C9C—N2C	11.94 (19)
C1B—C2B—C3B—F1B	−179.15 (18)	C7C—C8C—C9C—C10C	−108.26 (18)
C1B—C2B—C3B—C4B	2.1 (3)	N2C—C9C—C10C—C15C	−80.1 (2)
F1B—C3B—C4B—C5B	178.73 (18)	C8C—C9C—C10C—C15C	34.1 (2)
C2B—C3B—C4B—C5B	−2.5 (3)	N2C—C9C—C10C—C11C	98.58 (19)
C3B—C4B—C5B—C6B	0.2 (3)	C8C—C9C—C10C—C11C	−147.21 (17)
C4B—C5B—C6B—C1B	2.3 (3)	C15C—C10C—C11C—C12C	0.1 (3)
C4B—C5B—C6B—C7B	−178.54 (18)	C9C—C10C—C11C—C12C	−178.56 (19)
C2B—C1B—C6B—C5B	−2.7 (3)	C10C—C11C—C12C—C13C	−0.2 (4)
C2B—C1B—C6B—C7B	178.12 (17)	C11C—C12C—C13C—C14C	0.2 (4)
N2B—N1B—C7B—C6B	−179.56 (15)	C11C—C12C—C13C—F2C	−179.2 (2)
N2B—N1B—C7B—C8B	−0.1 (2)	C12C—C13C—C14C—C15C	−0.1 (4)
C5B—C6B—C7B—N1B	10.3 (3)	F2C—C13C—C14C—C15C	179.2 (2)
C1B—C6B—C7B—N1B	−170.53 (17)	C13C—C14C—C15C—C10C	0.1 (3)
C5B—C6B—C7B—C8B	−169.09 (19)	C11C—C10C—C15C—C14C	−0.1 (3)
C1B—C6B—C7B—C8B	10.1 (3)	C9C—C10C—C15C—C14C	178.58 (18)
N1B—C7B—C8B—C9B	3.8 (2)	N1C—N2C—C16C—O1C	178.61 (17)
C6B—C7B—C8B—C9B	−176.74 (18)	C9C—N2C—C16C—O1C	2.7 (3)
C16B—N2B—C9B—C10B	68.4 (2)	N1C—N2C—C16C—C17C	−1.3 (3)
N1B—N2B—C9B—C10B	−117.15 (16)	C9C—N2C—C16C—C17C	−177.25 (19)

### Hydrogen-bond geometry (Å, °)

**Table:**

*D*—H···*A*	*D*—H	H···*A*	*D*···*A*	*D*—H···*A*
C12A—H12A···F2A^i^	0.93	2.53	3.294 (3)	139
C12B—H12B···F2C^ii^	0.93	2.53	3.309 (3)	141
C12C—H12C···F2B^iii^	0.93	2.53	3.320 (3)	143
C17A—H17A···F1C^iv^	0.96	2.53	3.276 (8)	134
C17B—H17D···F1A^v^	0.96	2.47	3.309 (4)	146
C17C—H17G···F1B^i^	0.96	2.55	3.311 (2)	137
C15A—H15A···O1C	0.93	2.32	3.225 (3)	165
C15B—H15B···O1A^vi^	0.93	2.36	3.262 (3)	162
C15C—H15C···O1B^vii^	0.93	2.42	3.281 (3)	153

Symmetry codes: (i) −*x*+1, −*y*, −*z*+1; (ii) *x*+1, *y*, *z*; (iii) *x*−1, *y*, *z*; (iv) *x*, *y*−1, *z*; (v) −*x*+2, −*y*+1, −*z*+1; (vi) −*x*+2, −*y*, −*z*+1; (vii) −*x*+1, −*y*+1, −*z*+1.
